# Two-Color-Thermography for Temperature Determination in Laser Beam Welding of Low-Melting Materials

**DOI:** 10.3390/s23104908

**Published:** 2023-05-19

**Authors:** Karen Schwarzkopf, Richard Rothfelder, Michael Rasch, Michael Schmidt

**Affiliations:** 1 Institute of Photonic Technologies, Friedrich-Alexander-Universität Erlangen-Nürnberg, Konrad-Zuse-Str. 3/5, 91052 Erlangen, Germanymichael.rasch@lpt.uni-erlangen.de (M.R.); michael.schmidt@lpt.uni-erlangen.de (M.S.); 2 Graduate School in Advanced Optical Technologies, Friedrich-Alexander-Universität Erlangen-Nürnberg, Paul-Gordan-Str. 6, 91052 Erlangen, Germany

**Keywords:** two-color-thermography, temperature, temperature determination, ratio-based temperature measurement, laser beam welding, low-melting materials

## Abstract

Spatial and temporal knowledge of temperature evolution is crucial in laser beam welding of low-melting materials such as aluminum alloys. Current temperature measurements are restricted to (i) one-dimensional temperature information (e.g., ratio-pyrometers), (ii) a priori knowledge of emissivity (e.g., thermography), and (iii) high-temperature regions (e.g., two-color-thermography). This study presents a ratio-based two-color-thermography system that enables acquiring spatially and temporally resolved temperature information for low-melting temperature ranges (<1200 K). The study demonstrates that temperature can be accurately determined despite variations in signal intensity and emissivity for objects emitting constant thermal radiation. The two-color-thermography system is further transferred into a commercial laser beam welding set-up. Experiments with varying process parameters are conducted, and the ability of the thermal imaging method to measure dynamic temperature behavior is assessed. Image artifacts presumably caused by internal reflections inside the optical beam path limit the direct application of the developed two-color-thermography system during dynamic temperature evolution.

## 1. Introduction

Laser beam welding of metals is a thermal-driven joining process employing laser radiation as the heat source. By focusing the laser radiation on top of the sample surface, the metal is—depending on the laser intensity—transferred from a solid to a liquid phase (conduction mode welding) and/or gaseous phase (keyhole mode welding) due to absorption by the material. Compared to conventional welding methods, laser beam welding is characterized by a narrow heat-affected zone, increased processing speeds, and the ability to join a wide range of materials [1,2]. This is of high relevance, for example, in the automotive sector where the joining of low-melting aluminum alloys (such as the aluminum wrought alloys (e.g., AlMg4,5Mn and AlMgSi1) is considered a major challenge due to the associated hot cracking susceptibility.

Knowledge of spatial and temporal temperature evolution is crucial to understanding and controlling microscopic and macroscopic process phenomena in the laser beam welding of aluminum alloys. On the microscopic process level, inadequate heat input can provoke strong heterogeneity of the microstructure (e.g., precipitation for Al-alloys), which in turn favors heterogeneity of the mechanical properties [3]. On the macroscopic process level, inadequate heat input can provoke an unstable melt pool behavior which in turn favors, for example, spatter formation, melt pool expulsion, and porosity due to a collapsing keyhole [4]. Being able to determine spatial and temporal temperature information during laser beam welding of low-melting materials is, therefore, fundamental to (i) enhance process understanding, (ii) increase process efficiency, and (iii) derive possible control approaches.

### Temperature Determination in Laser Beam Welding

In laser beam welding, mainly optical approaches are employed for temperature determination. While pyrometric methods generally provide pointwise (one-dimensional) temperature information, thermographic methods can also be used to determine temperature information with spatial resolution. Classical pyrometric or thermographic approaches depend on prior knowledge of the emissivity for correct temperature determination. The emissivity ε is a dimensionless parameter that describes how well an object emits thermal radiation in comparison to an ideal black body radiator. The emissivity of an object depends on its material, temperature, phase, surface roughness, etc., and is only known to a limited extent [5]. Especially during laser material processing of metallic materials, significant variations of emissivity can be present [6]. For instance, the emissivity of die-casting iron is reported to vary between 0.45 (373 K) and 0.6–0.7 (1273 K) [7], and the emissivity for Inconel changes from 0.1 to 0.95 solely depending on the surface preparation [6]. Without the prior time-consuming experimental determination of the emissivity, only the so-called brightness temperature can be determined using classical pyrometry and thermography [8,9], which may not match the actual surface temperature with sufficient accuracy [5].

Ratio-based temperature determination methods are based on the collection of the emitted thermal energy in two closely spaced spectral ranges at the same time. Temperature can be determined independently of local emission variations, provided that the intensity ratio of the detected spectral radiance changes constantly [10,11]. For a detailed explanation of the physical background, the reader is referred to Section 2.1.1.

With two-color-pyrometry, a ratio-based, one-dimensional temperature determination method is already established in laser material processing. Furumoto et al. record the surface temperature in a self-designed additive manufacturing set-up using two-color pyrometry and demonstrate that the consolidation behavior of the powder particles depends on the introduced energy density [12]. Gutknecht et al. demonstrates under real process conditions that the signal from a two-color pyrometer correlates with the process stability [13]. Mitchell et al. demonstrate that there is a direct correlation between in situ sensed temperature signatures and occurring porosity in laser-based powder bed fusion of metal (PBF-LB/M) alloys [14]. While ratio pyrometers allow sufficiently high temporal resolution of the melt pool temperature, they are not suitable for determining spatially resolved temperature information [15].

Two-color-thermography combines the advantages of two-color-pyrometry and thermography. Similar to the one-dimensional two-color-pyrometry approach, thermal radiation is separated into two narrow wavelength ranges but captured with a two-dimensional detector element, for example, by identical camera sensors. By overlapping the signal intensity of individual detector elements congruently, the intensity ratio can be calculated pixel by pixel, and temperature can be determined with spatial resolution despite local emissivity variations. To date, this approach has been used only sporadically in the context of the laser material process. Hooper presents for the first time a coaxially guided two-color camera set-up for temperature determination in the PBF-LB/M process for the purpose of process optimization [15]. The detector material used is sensitive between 450 and 1000 nm, which corresponds to a temperature range of approximately 1000–4000 K. With a highly resolved acquisition (100 kHz acquisition rate, 20 μm spatial resolution), the temperature gradients on the melt pool level can be recorded as a function of process, geometry, and position parameters for Ti6Al4V. However, memory capacity limits the acquisition time to 3 s restricting the temperature acquisition to a single layer. The coaxially guided two-color thermography system developed by Vallabh et al. allows the thermal radiation emitted from the process zone to be combined into a single camera sensor via two differently filtered beam paths [16]. The measurement system is characterized by an acquisition rate of 30 kHz at a spatial resolution of 20 μm and demonstrates for the first time that two-dimensional, ratio-based temperature acquisition can be implemented over multiple layers. The lowest temperature that can be detected by the system is reported to be 1300 K.

Currently, no measurement method is available that allows one to determine spatial and temporal temperature information independent of local emissivity variations in laser material processing of low-melting metals (<1200 K). As common materials such as AlMg4,5Mn (T_Melt_ = 911 K) and AlMgSi1 (T_Melt_ = 923 K) are susceptible to defect formation, recording their thermal process behavior during laser beam welding is essential to further examine defect formation phenomena as well as to identify potential control approaches. For instance, laser beam welding of AlMgSi1 is highly susceptible to hot cracking due to a low silicon and magnesium content (about 1%). In contrast, laser-based welding of AlMg4,5Mn has a reduced susceptibility to hot cracking due to a high magnesium content (about 4.5%), but the high evaporation rate fosters process instabilities that lead to spatter formation, melt pool expulsion, and porosity [4].

This study presents a temperature measurement set-up based on two-color thermography that can determine spatial and temporal temperature information below 1200 K. The experimental set-up enables accurate temperature determination for objects emitting constant thermal radiation despite variations in signal intensity and emissivity. Within this study, the two-color thermography approach is, for the first time, integrated into an industrial laser welding machine to evaluate its performance under dynamic process conditions.

The paper is structured as follows: the physical background and the experimental set-up of the two-color-thermography system are presented in the material and methods chapter, followed by the calibration and image processing methods used. To prove the working principle of the two-color-thermography set-up, the methodology to test the system’s response towards signal intensity and emissivity changes at constant temperatures is further described. Last, the integration of the two-color-thermography system into an industrial laser beam welding set-up is described in the material and method chapter. The subsequent chapter presents the results of the conducted experiments, followed by a discussion of the results in the fourth chapter. The paper ends with a conclusion section that summarizes all findings and gives an outlook of future work approaches.

## 2. Materials and Methods

The following chapter is divided into five subsections. In the first section, the theoretical background of ratio-based temperature determination is explained. This is followed by the presentation and calibration of the developed two-color-thermography system. In the next section, the image processing steps are described to derive temperature information from individual images. Next, the methodology to prove the invariance of the two-color-thermography system towards signal intensity and emissivity changes at constant temperatures is described. This is followed by the last section, which focuses on the application of the two-color-thermography system in an industrial laser beam welding machine.

### 2.1. Two-Color-Thermography

#### 2.1.1. Physical Background

All objects with a temperature greater than absolute zero emit thermal radiation. The spectral radiance *M* (W·sr^−1^·m^−3^) that is emitted by an ideal black body for wavelength *λ* at absolute temperature *T* is given by Planck’s radiation law:

(1) $$M_{\lambda }\left(\lambda ,T\right)=\frac{2\pi hc^{2}}{\lambda^{5}}\cdot \frac{1}{e^{\frac{hc}{\lambda k_{B}T}}-1}$$

where *h* is the Planck constant, *c* is the speed of light, and *k_B_* is the Boltzmann constant. With an increase in temperature, the absolute amount of emitted energy of an object increases while the peak of the emitted spectrum shifts to smaller wavelengths.

Real objects do not emit thermal radiation perfectly. To describe how well an object emits thermal radiation in comparison to an ideal black body radiator, the dimensionless parameter emissivity ε is introduced. The emissivity of an object depends on its material, temperature, phase, surface roughness, etc., and is only known to a limited extent [5]. Especially during laser material processing of metallic materials, significant variations of emissivity can be present [6]. These process-induced emissivity variations hinder the direct application of Planck’s law for temperature determination based on a single wavelength in laser material processing. One approach to overcome this limitation is by simultaneous measure the spectral radiance at two narrow but separated spectral bands.

By measuring the thermal radiation of an object within two spectral bands (*λ*_1,*min*_–*λ*_1,_*_max_*, *λ*_2,_*_min_–λ*_2,_*_max_*), two signal intensities, *I*_1_ and *I*_2_, are determined, and the following intensity ratio *r* can be determined:

(2) $$r=\frac{I_{1}}{I_{2}}=\frac{\int_{\lambda 1,min}^{\lambda 1,max}\eta_{1}\cdot \varepsilon_{1}\left(\lambda_{1},T\right)\cdot M_{\lambda ,1}\left(\lambda_{1},T\right)\cdot d\lambda }{\int_{\lambda 2,max}^{\lambda 2,max}\eta_{2}\cdot \varepsilon_{2}\left(\lambda_{2},T\right)\cdot M_{\lambda ,2}\left(\lambda_{2},T\right)\cdot d\lambda }\;\;$$

where η is the combined efficiency of the optical path and the quantum efficiency of the detector (here: based on indium gallium arsenide (InGaAs)). Under the assumption that the chosen wavelength regions are close to each other and that the relative change in the emissivity is constant, it can be assumed that *ε*_1_~*ε*_2_. Then, the formula can be noted as follows:

(3) $$r=\frac{I_{1}}{I_{2}}=\frac{\int_{\lambda 1,min}^{\lambda 1,max}\eta_{1}\cdot M_{\lambda ,1}\left(\lambda_{1},T\right)\cdot d\lambda }{\int_{\lambda 2,max}^{\lambda 2,max}\eta_{2}\cdot M_{\lambda ,2}\left(\lambda_{2},T\right)\cdot d\lambda }$$

If the change in emissivity affects both spectral ranges to the same extent, the temperature can be determined, eliminating any error from emissivity variation [6]. The validity of this assumption is given in the work of [17]. It is shown that the emissivity of pure aluminum declines to the same extent towards higher wavelengths for different temperatures in the wavelength range between 0.8 and 2.2 µm. The validity is further underlined by research studies that employ the same ratio-based temperature measurement approach in the context of laser material processing, such as [13,15,16]. Under the gray body assumption, an emissivity-independent expression of temperature *T* can be noted:

(4) $$T=\frac{k_{B}\cdot \left(\lambda_{1}-\lambda_{2}\right)}{\lambda_{1}\cdot \lambda_{2}\cdot ln{\left(r\cdot \frac{\lambda_{1}}{\lambda_{2}}\right)}^{5}}$$

#### 2.1.2. Experimental Set-Up and Combined Optical Path Efficiency

**Figure 1:**
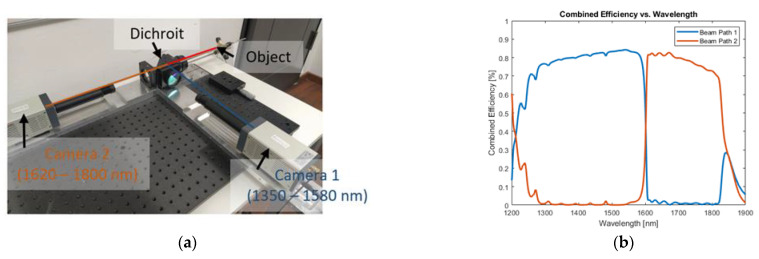
(**a**) Experimental set-up of the two-color-thermography set-up. The system employs two identical but spectrally differently filtered InGaAs-sensor modules (C15370-01, Hamamatsu Photonics Deutschland GmbH, Herrsching, Germany). (**b**) Simulated combined efficiency along the two spectral differently filtered beam paths (blue: beam path 1 (spectral band: 1350–1580 nm), red: beam path 2 (spectral band: 1620–1800 nm)) plotted against the wavelength.

The two-color-thermography set-up combines two identical InGaAs-sensors (C15370-01, Hamamatsu Photonics Deutschland GmbH, Herrsching, Germany) with an optical lens and filter system, as shown in Figure 1a. All optical elements are attached to a customized mounting plate to enable lateral integration of the measurement set-up into an industrial laser welding machine. The thermal radiation from the laser welding process zone is directed via an achromat (f = 250 mm, AC508-250-C-ML, Thorlabs Inc., Newton (NJ), USA) onto a dichroic beamsplitter (HR1030-1070HT800-1700, Laser Components Germany GmbH, Olching, Germany). Due to a customized optical coating, the dichroic beamsplitter is highly reflective in the wavelength range between 1350 nm and 1580 nm (beam path 1) and highly transmissive between 1620 nm and 1800 nm (beam path 2). The backfront of the dichroic mirror is coated with an additional anti-reflection coating minimizing reflectivity to <1%. After passing the spectral partition stage, the wavelengths of interest pass a second achromat (f = 250 mm, AC508-250-C-ML, Thorlabs) in each of the individual beam paths. In the final step, the thermal radiation is focused on the respective camera sensor by a lens (LF1547-C, Thorlabs). The simulated combined efficiency (multiplication of the optical path and the detector efficiency) is plotted against the wavelength for beam path 1 (blue) and beam path 2 (orange) in Figure 1b. For both beam paths, the combined efficiency is greater than 72% in the wavelength region of interest (gray). The employed InGaAs-sensors for temperature analysis allow recording speeds of up to 507 frames per second at a resolution of 320 × 256 pxl^2^ with a 16-bit pixel depth.

### 2.2. Calibration

**Figure 2:**
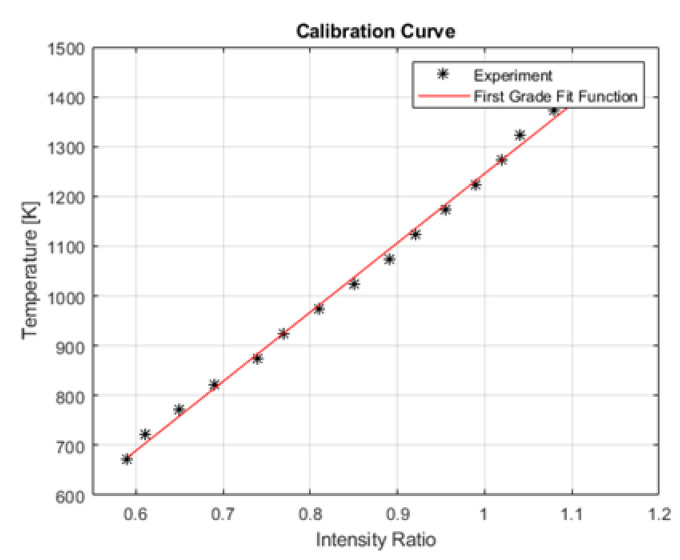
Calibration curve of the two-color-thermography system plotting the intensity ratio versus temperature. The slope of the experimentally determined data points can be approximated by the following linear equation: *T* = 1391 · r−145.

To correlate the ratio of the signal intensities with temperature, a calibration measurement based on a blackbody radiation source is conducted. The blackbody radiation system (Pegasus R Model 970, Isothermal Technology Ltd., Southport, UK) is housed—with suitable insulation—in a tube furnace. The cavity of the tube furnace measures 65 mm in depth and 20 mm in diameter, and its emissivity is specified at 0.995 [18]. The temperature of the furnace is set via a control panel, while the actual radiation temperature is measured by a thermocouple that is additionally inserted into the cavity. The temperature range of the blackbody radiation lies between 423 K and 1473 K. To collect the signal intensity of the individual camera sensors at a defined temperature, the two-color-thermography system is coaxially aligned to the cavity aperture of the blackbody radiator system. The focal plane of the system is positioned approximately in the middle of the cavity depth. A calibration curve ranging between 673 K and 1450 K (step size = 50 K) is derived. At each temperature plateau, the signal intensity is determined by averaging the signal intensity values of 50 consecutive frames for both camera sensors. In Figure 2, the determined calibration curves plot the intensity ratio versus the temperature. The experimentally determined data points (black stars) have a linear slope that can be approximated by the following linear equation (red line)

(5) T=1391·r−145

where *T* is temperature and *r* is the intensity ratio.

### 2.3. Image Processing

**Figure 3:**
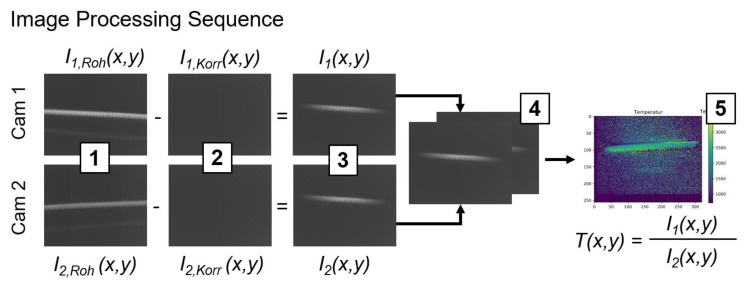
Exemplary sequence of image processing steps for temperature determination with the two-color-thermography system. For the sake of visualization, an incandescent filament is used as a radiation source as the camera sensors are not sensitive at ambient temperatures. The image processing sequence consists of (**1**) raw images, (**2**) dark-frame images, (**3**) noise-corrected images, (**4**) image alignment, and (**5**) calculation of intensity ratio and temperature assignment.

This section presents the image processing method used to obtain temperature data from individual images. The camera sensors are not sensitive at ambient temperature, therefore, an incandescent filament is used as a radiation source for the sake of visualization of the image processing sequence plotted in Figure 3. It shall be noted that all experiments presented in this paper employ the full sensor area (320 × 256 pxl^2^), which equals a measurement area of approx. 3.4 × 3.8 mm^2^. The frame rate is constantly set to 507 fps. To minimize distortion caused by thermal noise, the InGaAs-sensors are constantly cooled to a temperature of 253 K during operation. Figure 3 visualizes the five image processing steps that are applied:

1. Initial raw images:

*I*_1,*Roh*_(*x*,*y*), *I*_2,*Roh*_(*x*,*y*)

2. Subtraction of dark-frame images from raw images:

*I*_1,*Roh*_(*x*,*y*)—*I*_1,*Korr*_(*x*,*y*), *I*_2,*Roh*_(*x*,*y*)—*I*_2,*Korr*_(*x*,*y*)

3. Noise-corrected images (elimination of dark current and fixed-pattern noise):

*I*_1_(*x*,*y*), *I*_2_(*x*,*y*)

4. Image alignment based on a similarity measure (cross-correlation)
5. Calculation of intensity ratio and temperature assignment for each pixel

### 2.4. Proof-of-Concept at Constant Temperature

Prior to the adaption of the two-color-thermography system into a highly dynamic process environment (e.g., laser beam welding), the working principle shall first be proved for simple process conditions. Therefore, a non-moving object emitting constant thermal radiation (=constant temperature) is used. To systematically evaluate the ability of the thermal imaging system despite changes in signal intensity and emissivity, the following two research hypotheses are formulated:

1. A variation of signal intensity does not affect the temperature determination based on the two-color-thermography system.
2. A variation of emissivity does not affect the temperature determination based on the two-color-thermography system.

To evaluate the first research hypothesis, the black body radiator from the calibration procedure is used. The thermal radiation emitted by the black body radiator is set to 1173 K. Having set a constant temperature, the exposure times of the two camera sensors are consecutively varied between 5 µs and 10 µs. By employing a higher exposure time, the amount of thermal radiation collected by the semiconductor material increases. The amount of thermal radiation collected by the semiconductor material, in turn, decreases by employing a lower exposure time. It is expected that the variation of exposure time affects the thermal radiation collected in spectral band 1 to the same extent as the thermal radiation collected in spectral band 2. This, in turn, means that their ratio is constant. In accordance with research hypothesis 1, the same average temperature should be determinable despite changing the exposure time.

**Figure 4:**
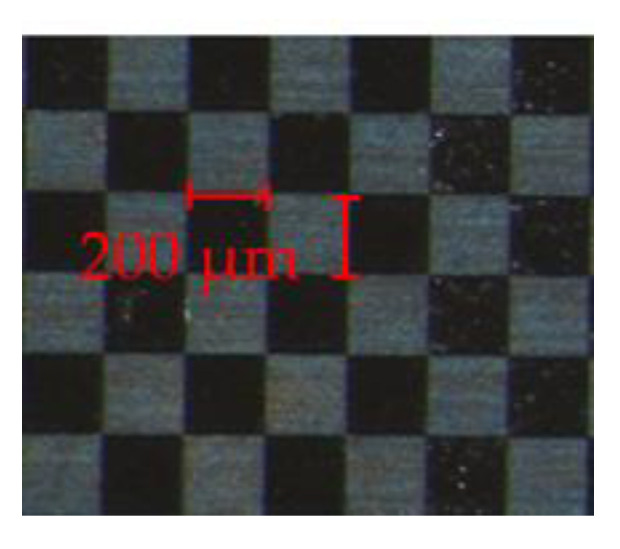
Top-view of a laser-marked checkerboard that is used to prove that the two-color-thermography system is insensitive to emissivity changes. The emissivity of the dark and bright tiles differs.

To evaluate the second research hypothesis, a checkerboard (7 × 6 tiles) made of stainless steel with an edge length of 200 µm is placed on a heating plate. The custom-made checkerboard is laser-marked and displayed in Figure 4. The surface structure of the laser-marked tiles is different compared to the unmarked tiles reasoning their darker appearance. The change in surface appearance comes with a change in emissivity resulting in a metal surface with varying emissivity properties. To evaluate the second research hypothesis, the checkerboard is heated over a wide range of temperatures by manually adjusting the heating plate. The pre-defined temperature is displayed on the control screen provided by the heating plate. As the unmarked and laser-marked tiles have different emissivity properties, the intensity of the thermal radiation directed toward the InGaAs-sensors is expected to vary. According to the second research hypothesis, it is expected that the calculated mean temperature is constant despite emissivity variation.

### 2.5. Experimental Set-Up for Laser Beam Welding

**Figure 5:**
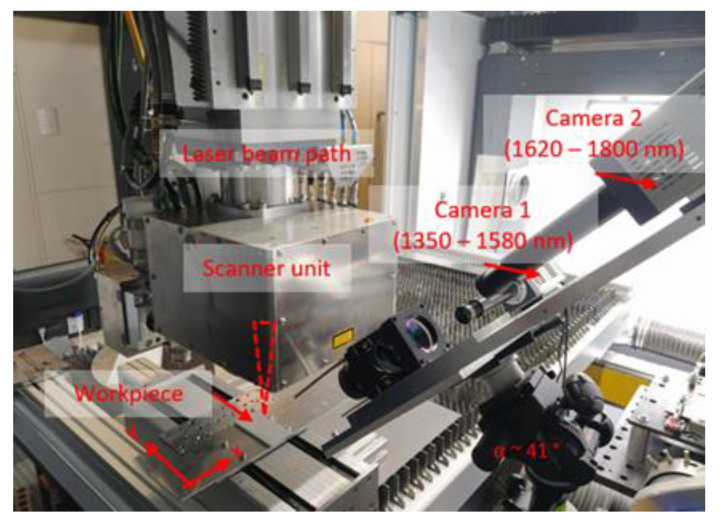
Off-axis integration of the two-color-thermography set-up into the industrial laser welding machine. The laser beam is guided in an x-direction across the workpiece with a scanner unit. The laser-matter-interaction zone is simultaneously observed with the two-color-thermography set-up. The set-up is inclined by approximately 41° relative to the workpiece surface.

To investigate the ability of the developed two-color-thermography system under real laser beam welding conditions, the experimental set-up is integrated into an industrial laser welding and cutting machine (ERLASER UNIVERSAL, ERLAS Erlanger Lasertechnik GmbH, Erlangen, Germany). Using the two-color-thermography set-up, it is aimed to identify temperature-related process characteristics (e.g., cooling rate, thermal gradient) and correlate them with the process result (e.g., melt pool depth and width). The integration of the thermal imaging system into the industrial laser beam welding system is shown in Figure 5. The laser beam welding machine consists of an enclosed housing containing the work area, a welding scanner unit, and a laser source provided by Trumpf GmbH. The disc laser TruDisk 8001 (λ = 1.030 nm) has a maximum output power of 8 kW. During the experiments, the laser beam is focused through the processing optics onto the surface of a metal sheet (dimension: 50 × 100 × 5 mm^3^, material: AlMg4,5Mn) and guided along the *x*-axis along the workpiece. The focus diameter is approximately 100 µm. The laser power (P) and the scan velocity (v) are varied between 200 W and 500 W and 100 mm/s and 500 mm/s, respectively. The two-color-thermography system is laterally focused on the workpiece with an inclination of approx. 41°. The exposure time of the InGaAs-cameras is set to 1 µs. The two-color-thermography set-up is manually triggered before and after the laser radiation is emitted.

## 3. Results

This chapter comprises two subsections. The first subsection demonstrates the legitimacy of the two-color-thermography system under process conditions employing constant temperature behavior. The thermal imaging system proves to be insensitive to changes in signal intensity and emissivity under these circumstances for temperature determination. The second subsection presents the findings from the laser beam welding experiments with dynamic temperature evolution using a commercial laser beam welding machine.

### 3.1. Proof-of-Concept at Constant Temperature

#### 3.1.1. Influence of Signal Intensity Variation

**Figure 6:**
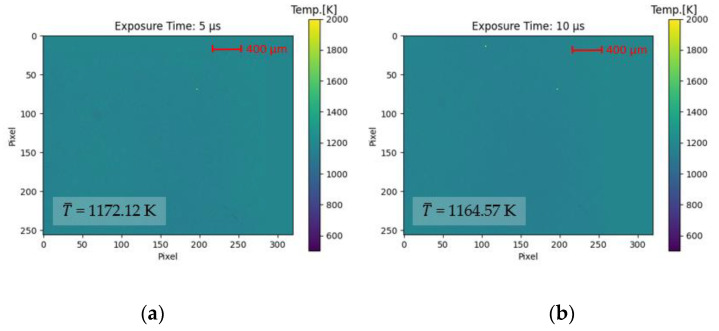
Influence of signal intensity on temperature determination by varying exposure time. The temperature is set to 1173 K while the exposure time is changed from (**a**) t_exp_ = 5 µs to (**b**) t_exp_ = 10 µs. Despite varying the signal intensity, temperature values can be determined with high accuracy (deviation <0.001% for 5 µs and −0.007% for 10 µs).

In this section, the results of the first research hypothesis are presented, which states that a variation of the signal intensity (amount of energy that is collected by the camera sensors) does not affect the temperature measurement based on the two-color-thermography system. In Figure 6, the calculated thermograms for the low and the high exposure time are compared against the defined temperature of 1173 K. On the left side (Figure 6a), the spatial temperature distribution for the lower exposure time of 5 µs is plotted against the sensor area (320 × 260 pxl^2^). The thermogram on the right side (Figure 6b) shows the spatial temperature distribution for the higher exposure time of 10 µs.

For the lower exposure time (5 µs), a mean temperature of 1172.12 K is calculated, whereas a mean temperature of 1164.57 K is determined for the higher exposure time (10 µs). The deviation from the defined temperature accounts for less than 0.001% (1172.12 K/1173 K) for the lower exposure time and −0.007% (1164.57 K/1173 K) for the higher exposure time. This is comparably low in comparison to reported measurement accuracies of ±1% for commercially available thermography systems. However, a direct comparison of the two-color-thermography system with commercially available devices has to be discussed cautiously at this early stage of development, especially because the determined temperature is an average value. Still, the ability of the two-color-thermography system to determine temperature with high accuracy independent of signal intensity changes for process conditions employing constant thermal radiation is demonstrated. This makes the two-color-thermography approach insensitive to, for example, process-induced intensity fluctuations in laser welding caused by contaminated protective glasses. From this follows that the first research hypothesis is confirmed.

#### 3.1.2. Influence of Emissivity Variation

**Figure 7:**
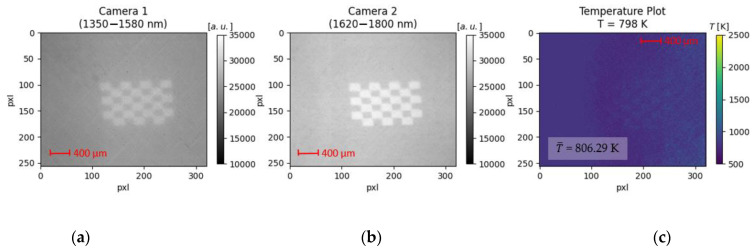
Influence of emissivity on temperature determination for process conditions employing constant thermal radiation. A checkerboard is placed on a heating plate and heated to 798 K. The thermal radiation emitted by the unmarked and laser-marked tiles differs, leading to a lower signal intensity for the unmarked and a higher signal intensity for the laser-marked regions. The checkerboard is observed with Camera 1 (**a**) and Camera 2 (**b**). The mean temperature (806.29 K) of the processed ratio-image deviates less than 0.01% from the defined temperature (798 K) (**c**).

In this section, the results of the second research hypothesis are presented, which states that a variation in emissivity does not affect the temperature determination based on the two-color-thermography system. The custom-made checkerboard used to imitate changing emissivity conditions is illustrated in Figure 7. The plot consists of three subplots, namely the raw images from Camera 1 (1350–1580 nm (Figure 7a)) and Camera 2 (1620–1800 nm (Figure 7b)) as well as the calculated thermogram (Figure 7c) for the given temperature of 798 K.

For the raw images, the signal intensity is plotted against the full sensor area. In both images, the checkerboard and its individual tiles with varying emissivity (laser-marked: bright tiles, unmarked: dark tiles) are visible. It can be noted that for the spectral region comprising smaller wavelengths (Figure 7a), the signal intensity is globally viewed as lower (or: darker) compared to the spectral region comprising the higher wavelengths (Figure 7b). This is based on Planck’s radiation law, where the emitted spectral radiance at the given temperature of 798 K is lower for the smaller wavelength band. Therefore, less energy is collected by the sensor of Camera 1, resulting in a less bright appearance of the checkerboard. Based on the prior described image processing sequence, the two raw images are processed, resulting in the plotted thermogram (Figure 7c). Unlike the raw images, the checkerboard is not visually distinguishable within the thermogram. This also manifests in the determined mean temperature of 806.29 K. The mean temperature deviates from the defined temperature by 0.01% (806.29 K/798 K). The low deviation manifests the ability of the two-color-thermography system to determine temperature with high accuracy independent of emissivity changes for process conditions employing constant thermal radiation. Thus, the second research hypothesis is also confirmed.

Since both research hypotheses are confirmed, it is concluded that the presented two-color-thermography approach is a legitimate method for temperature determination despite variations in signal intensity and variation of emissivity for process conditions employing constant thermal radiation. For both experiments, the deviation from the defined temperature is below 0.01%. The next section presents the results from the experiments conducted in the industrial laser beam welding machine.

### 3.2. Dynamic Temperature Evolution during Laser Beam Welding

In this section, the results of the experimental trials in the commercial laser beam welding machine are shown. The main goal is to investigate the ability of the developed two-color-thermography system to measure dynamic temperature evolution during laser beam welding. Based on the two-color-thermography set-up, it is aimed to identify temperature-related process characteristics (e.g., cooling rate, thermal gradient) and correlate them with the process result (e.g., melt pool depth and width).

**Figure 8:**
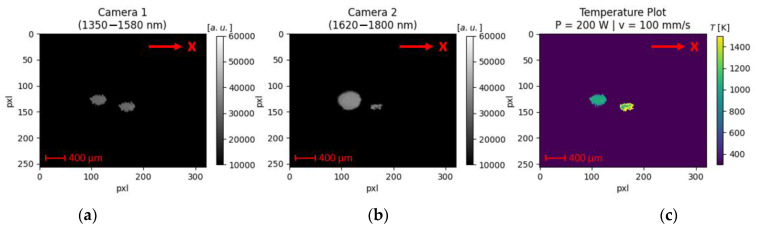
Example of image artifact present during laser beam welding trials (here: P = 200 W and v = 100 mm/s) in an industrial laser beam welding machine.

During all experiments with the industrial laser beam welding machine, a characteristic image artifact is notable. An exemplary visualization is shown in Figure 8a,b for experiments with a laser power of 200 W and a scan velocity of 100 mm/s. In both spectral bands, apparently, two laser-material interaction zones are present. However, it is physically impossible to have two laser-material interaction zones emitting toward the camera sensors, as only one laser beam source was used during the trials. This is confirmed in Supplementary Videos S1–S4, where the laser-interaction zone for the same parameter set was recorded using a high-speed imaging camera in combination with an illumination laser. It is clearly visible that the material is molten only at the position where the laser beam interacts with the sample surface.

Temperature determination under these circumstances is barely possible. First, it remains unclear which of the laser-material interaction zones is the original and which is the duplicate. Since the length (elongation along the x-direction) of the upper left object displayed in the thermogram (Figure 8c) conforms with the expected melt pool length (approx. 500 µm) for the chosen process parameters, it is concluded that this is the original laser-material interaction zone. Therefore, the right object in the resulting thermogram is invalid and will not be considered. The mean temperature of the right object is approx. 1000 K. From a physical perspective, this is reasonable, as the temperature value ranges between the melting (911 K) and evaporation (1883 K) temperature of the investigated material AlMg4,5Mn. However, there is no notable temperature variation between the center and the contours. A constant temperature evolution within the melt pool is doubtful, as a temperature decline from the center of the melt pool toward the edges is expected.

## 4. Discussion

### 4.1. Proof-of-Concept at Constant Temperature

With the developed two-color-thermography set-up, the temperature could be determined for objects emitting constant thermal radiation with high accuracy despite variations in (i) signal intensity and (ii) emissivity. Having the ability to temporally and spatially determine temperature independent of changes in signal intensity is of great benefit in laser beam welding. For instance, contaminated protective glasses or process fumes attenuate the signal that is directed toward the sensor unit. Measurement methods (e.g., pyrometer, thermography) that cannot account for such signal attenuations will determine temperature values that are strongly deviating from the defined temperature value leading to wrong process assumptions. Especially the ability of the presented two-color-thermography system to measure the temperature evolution with high temporal and spatial resolution despite emissivity changes is a huge potential for laser material processing. As stated before, emissivity can change drastically for commonly used materials in laser-based manufacturing processes [6]. Having a tool that can determine temperature reliably despite emissivity changes is, therefore, of high relevance.

### 4.2. Dynamic Temperature Evolution during Laser Beam Welding

The two-color-thermography set-up was successfully transferred into an industrial laser beam welding machine to investigate its ability to measure the temperature under real process situations. The developed two-color-thermography set-up can only determine the temperature during laser beam welding to a very limited extent. Image artifacts in the form of duplicates of the laser-material interaction zone are present for all conducted process parameter sets. The ghost imaging phenomena can explain the occurring artifacts. The reason for this is internal (multi-) reflections from surfaces within the beam path [19]. Possible sources of error could be, for example, a misalignment of optical components or reflections caused at the backside of the dichroic mirror. Although the back of the dichroic mirror is coated with a special anti-reflection coating, on average, up to 1% of the incident light could be reflected. This might be unfavorable for dynamic process situations, but further investigations are necessary, and an optimization of the optical beam path design employing optical components (e.g., pellicle beamsplitters) specialized in eliminating the ghosting phenomena is intended.

## 5. Conclusions

The main conclusions from the conducted investigations are summarized in the following:

1. A spatial and temporal temperature determination set-up based on the ratio principle is presented. The developed two-color-thermography set-up is designed to measure temperature in the region of low-melting materials (< 1200 K) based on InGaAs-sensors.
2. For process conditions employing constant thermal radiation, it is demonstrated that temperature determination is possible despite the signal intensity variation with the two-color-thermography system. The calculated mean temperature is reported to differ by <0.007% from the defined temperature despite doubling the exposure time of the camera sensors.
3. For process conditions employing constant thermal radiation, it is demonstrated that temperature determination is possible despite variation of emissivity with the two-color-thermography system. The calculated mean temperature of a custom-made checkerboard consisting of tiles with different emissivity deviated by less than 0.01% from the defined temperature value.
4. Image artifacts presumably caused by internal reflections inside the optical path of the two-color-thermography system restrict the direct transition of the set-up into a commercial laser welding machine.
5. Further investigations and optimization of the optical beam path design will be conducted to allow for reliable temperature measurement during the dynamic temperature evolution in laser beam welding of low-melting materials.

## Data Availability

The data presented in this study are available on request from the corresponding author.

## Supplementary Materials

The following supporting information can be downloaded at: https://www.mdpi.com/article/10.3390/s23104908/s1, Figure S1: Influence of signal intensity on temperature determination by varying exposure time; Figure S2: Influence of emissivity on temperature determination for process conditions employing constant thermal radiation; Figure S3: Example of image artifact present during laser beam welding trials; Video S1: Supplement_Video_200W_100mms; Video S2: Supplement_Video_200W_500mms; Video S3: Supplement_Video_500W_100mms; Video S4: Supplement_Video_500W_500mms.
