# Supplementary figures and images for: Two-Color-Thermography for Temperature Determination in Laser Beam Welding of Low-Melting Materials
Source: Sensors (Basel). 2023 May 19;23(10):4908. doi: 10.3390/s23104908 (PMC10220973; doi:10.3390/s23104908)

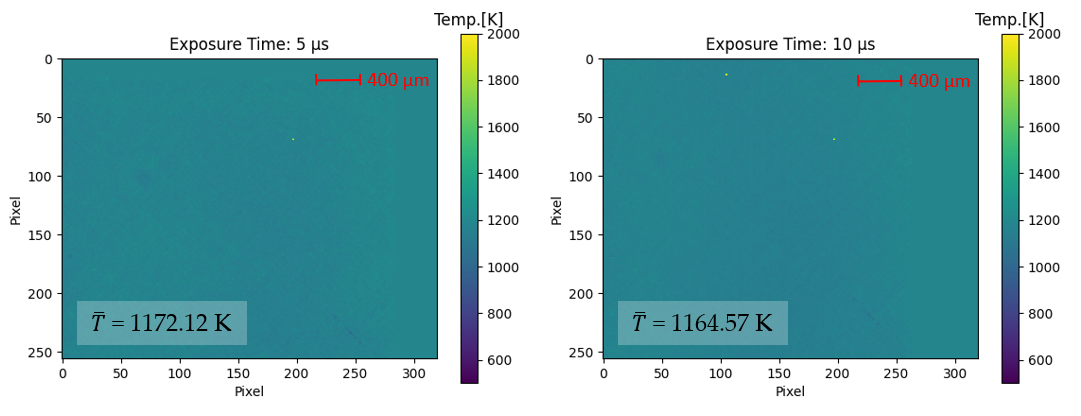

Supplement: Supplementary file 1 [file sensors-23-04908-s001.zip › S1_Fig. 6.png]

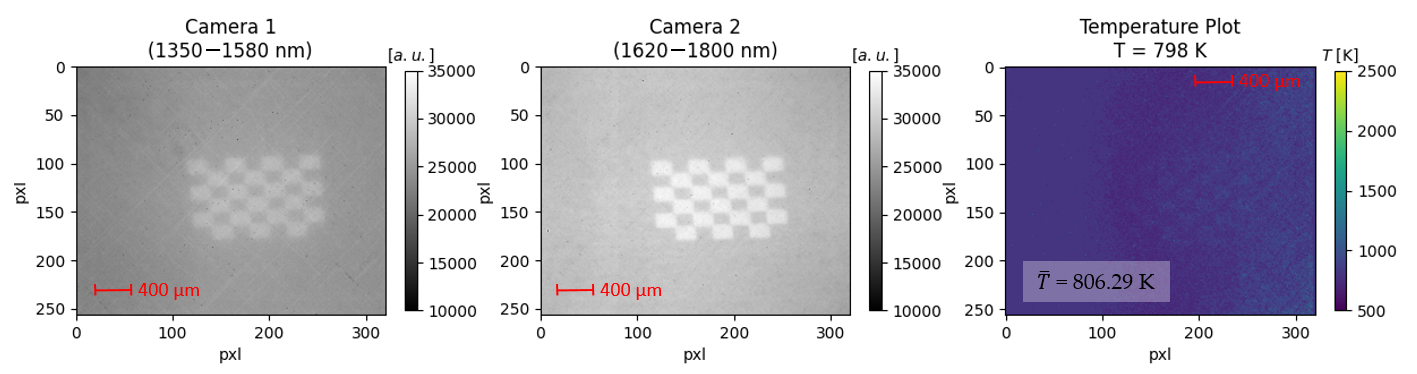

Supplement: Supplementary file 1 [file sensors-23-04908-s001.zip › S2_Fig. 7.png]

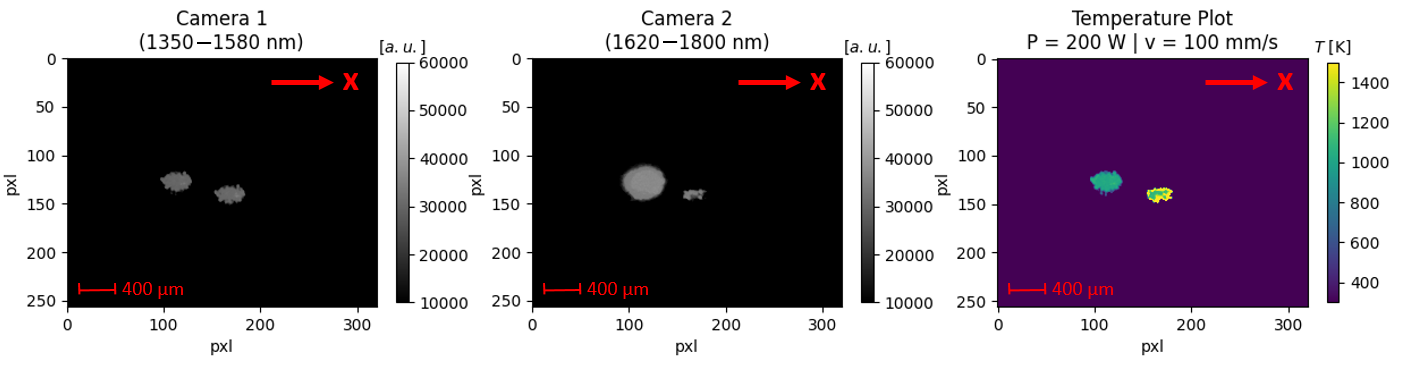

Supplement: Supplementary file 1 [file sensors-23-04908-s001.zip › S3_Fig. 8.png]
